# Temporal and palaeoclimatic context of the evolution of insular woodiness in the Canary Islands

**DOI:** 10.1002/ece3.7986

**Published:** 2021-08-17

**Authors:** Alexander Hooft van Huysduynen, Steven Janssens, Vincent Merckx, Rutger Vos, Luis Valente, Alexander Zizka, Maximilian Larter, Betül Karabayir, Daphne Maaskant, Youri Witmer, José María Fernández‐Palacios, Lea de Nascimento, Ruth Jaén‐Molina, Juli Caujapé Castells, Águedo Marrero‐Rodríguez, Marcelino del Arco, Frederic Lens

**Affiliations:** ^1^ Naturalis Biodiversity Center Leiden The Netherlands; ^2^ Department of Biology University of Antwerp Antwerp Belgium; ^3^ Meise Botanic Garden Meise Belgium; ^4^ Department of Biology KU Leuven Leuven Belgium; ^5^ Institute for Biodiversity and Ecosystem Dynamics University of Amsterdam Amsterdam The Netherlands; ^6^ Groningen Institute for Evolutionary Life Sciences University of Groningen Groningen The Netherlands; ^7^ German Center for Integrative Biodiversity Research (iDiv) Leipzig Germany; ^8^ Island Ecology and Biogeography Research Group Instituto Universitario de Enfermedades Tropicales y Salud Pública de Canarias Universidad de La Laguna (ULL) La Laguna Spain; ^9^ Jardín Botánico Canario “Viera y Clavijo”‐Unidad Asociada al CSIC (Cabildo de Gran Canaria) Las Palmas de Gran Canaria Spain; ^10^ Departamento de Botánica Ecología y Fisiología Vegetal Universidad de La Laguna (ULL) La Laguna Spain; ^11^ Institute of Biology Leiden, Plant Sciences Leiden University Leiden The Netherlands

**Keywords:** Canary Islands, drought, insular woodiness, molecular dating, palaeoclimate, wood formation

## Abstract

Insular woodiness (IW), referring to the evolutionary transition from herbaceousness toward woodiness on islands, has arisen more than 30 times on the Canary Islands (Atlantic Ocean). One of the IW hypotheses suggests that drought has been a major driver of wood formation, but we do not know in which palaeoclimatic conditions the insular woody lineages originated. Therefore, we provided an updated review on the presence of IW on the Canaries, reviewed the palaeoclimate, and estimated the timing of origin of woodiness of 24 insular woody lineages that represent a large majority of the insular woody species diversity on the Canaries. Our single, broad‐scale dating analysis shows that woodiness in 60%–65% of the insular woody lineages studied originated within the last 3.2 Myr, during which Mediterranean seasonality (yearly summer droughts) became established on the Canaries. Consequently, our results are consistent with palaeoclimatic aridification as a potential driver of woodiness in a considerable proportion of the insular woody Canary Island lineages. However, the observed pattern between insular woodiness and palaeodrought during the last couple of million years could potentially have emerged as a result of the typically young age of the native insular flora, characterized by a high turnover.

## Graphical abstract

We provided an updated review on the presence of insular woodiness on the Canaries, reviewed the palaeoclimate, and estimated the timing of origin of woodiness of 24 insular woody lineages of the Canary Islands (Spain). Our single, broad‐scale molecular dating analysis shows that woodiness in 60%–65% of the insular woody lineages studied originated within the last 3.2 Myr, consistent with palaeoclimatic aridification as a potential driver of woodiness.

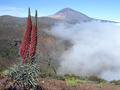

## 1 INTRODUCTION

Since the observations of Charles Darwin (1859) and Joseph Dalton Hooker (1867), marine islands have been regarded as ideal systems to unravel evolutionary processes that have shaped present‐day life due to their isolation and defined boundaries (Helmus et al., 2014; Losos & Ricklefs, 2009; Patiño et al., 2017). Despite comprising just 3.5% of the Earth's land area, marine islands—including volcanic islands, continental fragments, and land‐bridge islands—harbor up to 20% of all terrestrial species and thereby contribute disproportionately to global biodiversity (Whittaker et al., 2017). This implies that a large proportion of island species evolved through in situ speciation and are found nowhere else in the world, highlighting islands as natural laboratories of evolution. A famous example of the particularity of island biota is the repeated evolution of a peculiar suite of convergent traits in insular clades, often called “island syndromes”; examples are flightlessness in birds and insects, naïve behavior toward predators and body size changes across animals, and loss of dispersal ability, reduced defenses and insular woodiness in plants (Burns, 2019).

**FIGURE 1:**
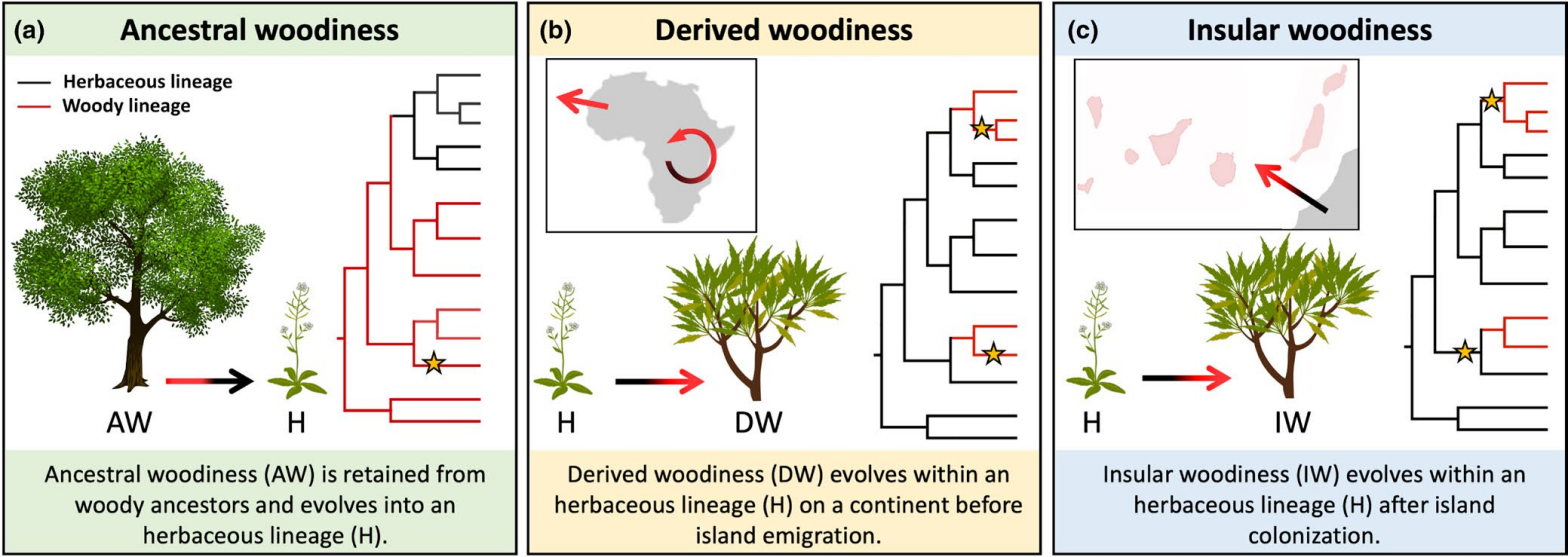
Explanatory graphic of ancestral woodiness (a), derived woodiness (b), and insular woodiness (c). The branches of the phylogenies are colored according to growth form (red = woody; black = herbaceous). Star symbols highlight the estimated time of island colonization and denote ancestral woody clades, derived woody clades, and insular woody clades in each panel, respectively

In flowering plants, woodiness is considered to be ancestral (Doyle, 2012; Feild et al., 2004), meaning that herbaceous lineages lost woodiness that characterized their ancestrally woody ancestors. Surprisingly, many herbaceous lineages gave rise to new woody species (shrubs or even trees) on islands and continents, an evolutionary reversal known as (phylogenetically) derived woodiness (Carlquist, 1974; Givnish, 1998; Lens et al., 2013). Insular woodiness (IW) in a strict sense—defined as the evolution of the woody growth form on (sub)tropical islands after arrival of herbaceous colonizers (Figure 1)—is the best‐known type of derived woodiness. This evolutionary phenomenon explains why these islands harbor a higher proportion of woody plant species than nearby continents, which is without doubt the most striking feature of (sub)tropical insular floras (Carlquist, 1974; Lens, Davin et al., 2013; Whittaker & Fernández‐Palacios, 2007).

Why have so many herbaceous lineages returned to a woody life form on islands? This key question remains at the forefront of island studies more than 50 years after the ground‐breaking publication of MacArthur and Wilson's *Theory of Island Biogeography* (Patiño et al., 2017). A number of hypotheses have been put forward to explain IW, such as (i) increased competition (Darwin (1859), elaborated by Givnish (1998): taxon‐cycling hypothesis), (ii) greater longevity (Wallace (1878); elaborated by Böhle et al. (1996): promotion‐of‐outcrossing hypothesis), (iii) favorable climate (especially lack of frost; Carlquist (1974)), and (iv) reduced herbivory (Carlquist, 1974); for a more detailed explanation of these hypotheses, see Whittaker and Fernández‐Palacios (2007). However, our current knowledge about IW is too fragmented to allow developing new hypotheses or rigorously testing existing ones, and the few known experimental tests are based on small‐scale examples (Givnish, 1982; Percy & Cronk, 1997). Therefore, an IW overview for the Canary Islands—a volcanic archipelago about 100 km off the coast of northwest Africa—was compiled based on published molecular phylogenetic insights (Lens, Davin et al., 2013). That paper identified 220 native insular woody species that resulted from 38 independent shifts and showed that most of these species typically grow in regions with recurrent drought cycles. This laid the foundation for a fifth hypothesis proposing drought as a potential driver of woodiness, which was later supported by experimental measurements in Canary Island Asteraceae and Brassicaceae demonstrating that woodier stems are better adapted to avoid drought‐induced gas embolisms inside water‐conducting cells compared to the stems of herbaceous (or less woody) relatives (Brodribb, 2019; Dória et al., 2018). Since drought‐induced embolisms can develop rapidly into lethal levels of hydraulic failure blocking the long‐distance water transport, the ability of plants to resist embolism formation is essential for survival during periods of water shortage (Brodribb et al., 2020; Choat et al., 2018; Lens, Tixier et al., 2013). Likewise, the link between increased stem woodiness/lignification and drought‐induced embolism resistance is also demonstrated in lineages outside the Canaries, such as *Arabidopsis* (Lens, Tixier et al., 2013; Thonglim et al., 2020) and across grasses (Lens et al., 2016). However, the current‐day distribution patterns of insular woody species on the Canaries and the above‐mentioned experimental results in several lineages do not necessarily mean that woodiness originated in drier palaeoclimatic episodes.

The first objective of this paper is to critically evaluate the occurrence of insular woody species and the number of IW shifts on the Canaries based on the most recent molecular phylogenies published after the first IW overview paper (Lens, Davin et al., 2013). Secondly, we aim to investigate whether the origin of woodiness among multiple insular woody lineages is consistent with periods of palaeoclimatic aridification for the Canary Islands. Therefore, we reconstructed the palaeoclimatic history of the archipelago based on literature, and we used the largest dated phylogenetic framework for angiosperms (Janssens et al., 2020) based on *rbcL* and *matK* sequences for ca 24,000 species to simultaneously date colonizations and origins of woodiness in 24 insular woody lineages; these 24 IW transitions representing 94% of the Canarian IW species diversity were subsequently positioned in the context of palaeoclimate. In addition, we provided dating estimates for the colonization events of 15 additional native lineages representing nine herbaceous lineages that remained herbaceous on the archipelago, two ancestrally woody lineages, and four woody lineages that developed their woodiness outside the Canary Islands.

## 2 MATERIALS AND METHODS

### 2.1 Identification of insular woody species and evaluation of number of insular woodiness shifts

An initial species list of insular woody species was presented in the first review of IW on the Canaries (Lens, Davin et al., 2013). In that first review paper, the woodiness of native Canary Island lineages was inferred to have originated through one of two exclusive processes. Either (i) the woody lineages are deeply nested into an exclusively woody clade and only have woody relatives, which is the ancestral state within flowering plants (ancestral woodiness) from which all extant herbaceous lineages have evolved (Figure 1); alternatively, (ii) woody insular lineages regained their woodiness after the arrival of herbaceous island colonizers (insular woodiness), or (iii) they are derived from woody island colonizers that regained their woodiness fairly recently on nearby continents before island colonization (derived woodiness; Figure 1). To extract the needed information to test these hypotheses, we searched for the most recently published molecular phylogenies with sufficient sampling of the woody Canary Island clade and their relatives and combined them with trait information on the life form (woodiness versus herbaceousness) via intensive screening of regional floras, field guides, taxonomic treatments, and the study of herbarium specimens and wood anatomical sections in some cases. The shifts toward insular woodiness were validated by ancestral state reconstructions (see below). Since we were only interested in uncovering drivers of transitions toward a woody growth form, we consider species with only a woody stem base as not sufficiently woody, and consequently, we discarded them from our species list (Kidner et al., 2016; Lens et al., 2012).

For the species database, we used the Leipzig Catalogue of Vascular Plants (Freiberg et al., 2020) to resolve the taxonomic status of the respective names (accepted/synonym). We then retrieved information on the species’ growth form, maximum height, preferred habitat and minimum and maximum elevation of occurrence, from taxonomic treatments and floras (Table S1). For the genus database, we listed the total number of species, the number of insular woody species, and the number of insular woody shifts for each genus based on the phylogenies we consulted (Table S2). For some lineages, we indicated a variable number of insular woody shifts, since the available phylogenies did not allow us to conclude whether or not an insular woody clade developed its woodiness on the Canaries or nearby islands.

An ancestral state reconstruction was used to validate the origin of woodiness for several Canary Island clades with multiple species. We used a discrete MCMC model with BayesTraits version 3.0.1 (Meade & Pagel, 2017) on small subclades of the constructed megaphylogeny that include the Canary Island species and their closest continental sister lineages (Figures S1–S12).

### 2.2 Palaeoclimatic reconstruction

**FIGURE 2:**
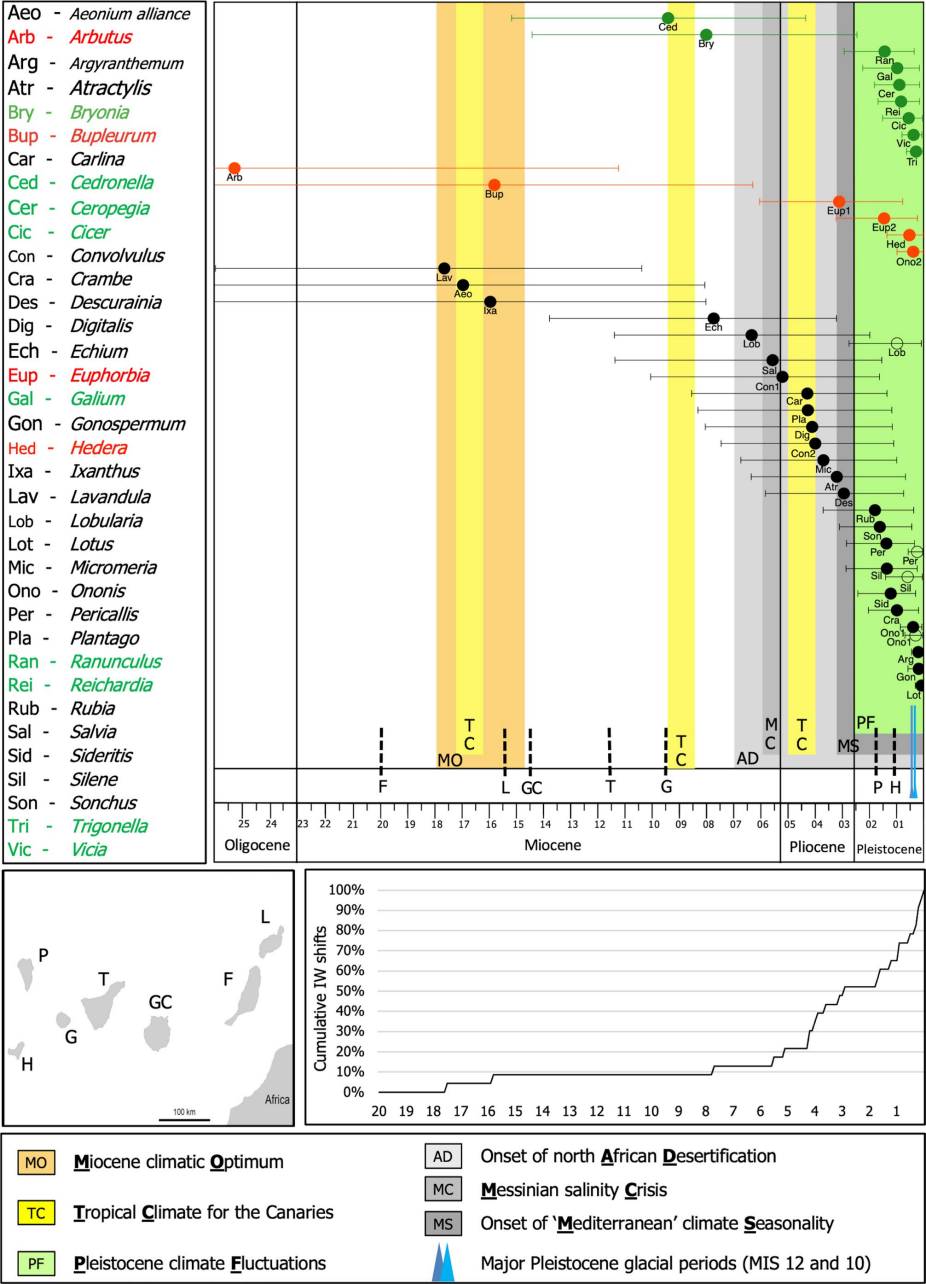
Timeline of 39 Canary Island colonization events and insular woody shifts based on conservative mean stem age estimates and their 95% range, along with the major palaeoclimatic events, ages of the individual Canary Islands over the last 26 Myr, and cumulative increase of insular woody shifts on the Canary Islands over the last 20 Myr. Black circles refer to the estimated mean ages for the timing of colonization of the insular woody clades; for *Lobularia*, *Ononis*, *Pericallis*, and *Silene*, which all include herbaceous as well as insular woody species on the Canary Islands, the insular woody shift occurred more recently in time (open circles). The time of colonization of the herbaceous clades are illustrated in green and the time of colonization of ancestrally woody clades and derived woody clades that have evolved their woodiness outside the archipelago are illustrated in red

A timeline of global, Mediterranean/North African, and Canary Island‐specific palaeoclimatic trends and events was compiled after a review of published literature (Figure 2, Table S3). Diverse sources of evidence were considered to reconstruct the palaeoclimate for the Canaries, including fossilized pollen, oceanic benthic foraminifera cores, results from geochemical or geological analyses, faunal trace fossils, and atmospheric models.

### 2.3 Marker choice and taxon sampling

The Consortium for the Barcode of Life working group (CBOL) recommended the two chloroplast markers *matK* and *rbcL* as primary barcodes to identify vascular plant species, resulting in massive amounts of sequences available in GenBank. The combination of both markers facilitates phylogenetic resolution in a complementary way: *rbcL* is a conservative locus useful for reconstructing deeper nodes, while *matK* contains rapidly evolving regions that can resolve shallower nodes (Hollingsworth et al., 2011; Janssens et al., 2020). Downside of this two‐gene approach is, however, that the resulting gene tree does not necessarily match the species tree, due to processes such as hybridization, incomplete lineage sorting, and horizontal gene transfer (Davidson et al., 2015). For this reason, we validated our dating results of native Canary Island lineages in two ways: (1) we only retained lineages for which the phylogenetic relationships between the Canary Island species and their closest continental relatives matched those found in previously published phylogenies based on additional markers, and (2) we compared the colonization times of native Canary Island lineages with previous dating estimates using different markers and fossil calibration points (Table S4).

Janssens et al. (2020) mined a considerable proportion of the available *matK‐rbcl* sequences from GenBank to compile an angiosperm‐wide dataset including 36,101 species, to which 56 fossils were assigned as calibration points. Since reliable fossils for insular woody lineages are lacking on the Canary Islands, this dataset allowed us to build an evolutionary framework in which we can compare the colonization times of the Canary Island lineages using a single analysis based on the same set of fossil calibration points and calibration settings. We modified the Janssens dataset to meet our research question: Eudicots were retained since most insular woody transitions on the Canaries belong to the asterid and rosid clades, and we excluded most of the early diverging angiosperms (no native insular woody lineages) as well as nearly all monocots (no wood development). This reduced our dataset to 23,776 species and an associated 42 fossil calibration points.

We then included *matK* and *rbcL* sequences from 137 Canary Island species that are endemic, native or probable native according to Arechavaleta et al. (2010). Of these species, 91 are insular woody according to the updated insular woody species list (Table S1). We also added *matK*‐*rbcL* sequences for 85 continental outgroup species (see Table S5) that were found to be most closely related to the native Canary lineages based on their position in previously published phylogenies. Of these 222 species in total, we generated original *matK* and *rbcL* sequences for 98 Canary Island species and 23 continental outgroup species collected from dried leaf samples or DNA samples via different institutes: (1) DNA Bank of the Canarian Flora and LPA herbarium, housed at the Botanical Garden ‘Viera y Clavijo’ ‐ Unidad Asociada al CSIC, Cabildo de Gran Canaria (Canary Islands, Spain), (2) herbaria of Naturalis Biodiversity Center (Leiden, The Netherlands), Meise Botanic Garden (Belgium), La Laguna University (Tenerife, Canary Islands, Spain), and Texas (USA), and (3) botanical gardens of Leiden (The Netherlands), Utrecht (The Netherlands), and Marburg (Germany). Sequences from the remaining species were downloaded from GenBank. Vouchers used in our study are compiled in Table S5.

Although we were targeting the closest related continental—mainly Mediterranean—species for each Canary Island lineage studied as identified by previous phylogenies (Table S5), it is possible that for several Canary Island lineages the closest relatives were not sampled in those phylogenies, have not yet been described, or have gone extinct. Therefore, the age estimates for island colonization should be considered as maximum ages and probably overestimate the true colonization time, especially when stem ages are used instead of crown ages. In our paper, we rely on stem age estimates (Figure 2) and crown node ages (Figure S13) to have a more balanced discussion on the colonization of island lineages (for a more detailed discussion, see García‐Verdugo et al., 2019).

In total, the species investigated belong to 39 independent, single colonization events and 18 flowering plant families: 24 insular woody lineages (representing 94% of the insular woody species diversity on the Canaries as identified in this paper), nine herbaceous eudicot lineages, four derived woody lineages (developed their woodiness outside the Canaries), and two ancestrally woody lineages. For the genus *Convolvulus*, two separate IW shifts were identified on the Canaries and were treated as independent IW events in our analysis (*Convolvulus* 1: *C*. *scoparius* versus *Convolvulus* 2: *C*. *canariensis*‐*C. fruticulosus*‐*C. lopez‐socasii*). Two woody Canary lineages of *Ononis,* one with an insular woody origin (*Ononis* 1: *O. christii*) and the other with a continental derived woody origin (*Ononis* 2: *O. hesperia*) were also dated independently (see Table S4). Furthermore, one *Euphorbia* lineage (*Euphorbia* 1: represented by *E. balsamifera*) has a known continental derived woody origin. A second independent woody *Euphorbia* lineage (*Euphorbia* 2: represented by *E. mellifera*) is included as a derived woody lineage because it is unclear whether the insular woody shift occurred on the Canaries or on adjacent Macaronesian archipelagos (Madeira, Azores).

For most of the nine native herbaceous Canary Island lineages, we selected clades from the same families that also represented the insular woody lineages. We removed a number of additional clades for which we generated sequences in our final analyses, because the *matK‐rbcL* phylogeny did not match the previously published phylogenies based on various plastid or nuclear markers. For instance, *matK‐rbcL* sequences did not discriminate between the Canary Island clade and the continental outgroup (e.g., *Canarina*, *Cheirolophus, Rumex,* and *Urtica*). For the *Aeonium* alliance, we could only provide the dating estimate for the colonization event, because the phylogenetic position of the insular woody *Aeonium* species were scattered among *Aichryson* and *Monanthes* species in our megaphylogeny. For the Canary Island *Lobularia*, *Convolvulus* 2 and *Ononis* 1 and 2 clades, *matK* and/or *rbcL* markers of the continental outgroup could not be successfully sequenced. Therefore, we selected the crown node to estimate colonization time (Table S4).

### 2.4 DNA extraction, sequencing protocols, and alignment

For each specimen, a 1 cm^2^ subsample of leaf tissue was obtained. The leaf material was lysed with sterile sand, and 7mm glass beads before DNA was extracted with the NucleoMag 96 plant kit (Macherey‐Nagel Gmbh & Co., Düren, Germany) using the KingFisher Flex magnetic particle processor (Thermo Scientific). The primer pair “1R‐KIM”/“3F‐KIM” (forward and reverse) was used for *matK* amplification (Hollingsworth et al., 2009). For *rbcL*, a 900 bp sequence was amplified with the forward primer “F1” and an internal reverse primer; another 800 bp sequence was amplified with an internal forward primer and the *rbcL* “85R” or “Z1375R” reverse primer (Kress & Erickson, 2007). Both DNA regions were amplified using an optimized PCR mixture consisting of 8.4 µl ultrapure H_2_O, 5.0 µl 5× PCR Phire reaction buffer, 1.0 µl 25 mM MgCl2, 1.0 µl 10 mg/ml BSA, 5 µl 100 mg/ml PVP, 0.5 µl 2.5 mM dNTPs, and 0.5 µl of Phire Hot Start II polymerase. A similar hot start PCR protocol was used for *matK* and *rbcL*, only differing in the annealing temperature (53°C and 66°C, respectively). Thermal cycling conditions were as follows: 98°C for 45 s (s), followed by 40 cycles of 98°C for 10 s, 53°C or 66°C for 30 s, 72°C for 40 s, and a final extension at 72°C for 7 min. The PCR products were verified by electrophoresis in 2% agarose e‐gel stained with SYBR™ Safe DNA Gel Stain (Thermo Scientific).

The PCR products were purified and sent to BaseClear (Leiden, The Netherlands) for bidirectional Sanger sequencing. Consensus sequences were generated and edited manually using Geneious version 11.1 (Kearse et al., 2012). The concatenated *matK* and *rbcL* sequences were added into an existing, aligned angiosperm‐wide dataset (Janssens et al., 2020). To facilitate the aligning process of our self‐generated sequences to the sequences already aligned in the dataset, we aligned each obtained sequence of a particular clade to a selection of phylogenetically related reference sequences in the existing dataset using MAFFT version 7 (with parameters keep alignment length: yes, progressive method: G‐INS‐i, all other parameters default) (Katoh & Standley, 2013). Manual adjustments were made using Geneious Prime. All the new DNA sequences have been deposited on GenBank with accession numbers MN783748–MN783978 (Table S5).

### 2.5 Construction of a dated maximum likelihood phylogenetic tree

We used the CIPRES scientific portal for maximum likelihood (ML) phylogeny inference using RAxML—HPC version 8 (Miller et al., 2010; Stamatakis, 2014). A rapid bootstrapping algorithm was implemented, examining 1,000 pseudo‐replicates. GTRCAT approximation was selected in accordance with RAxML recommendations for large trees following Izquierdo‐Carrasco et al. (2011); the other default parameters followed Stamatakis (2016). The phylogenetic relationships of all species in the broad‐scale aligned dataset were constrained at family level when conducting the RAxML analysis to (a) assure that the discriminatory power of *matK* and *rbcL* at the genus and species level does not come at the cost of correct deeper phylogenetic reconstruction and (b) to reduce computational time.

We took 42 age constraints across the angiosperm phylogeny from Janssens et al. (2020) (see Table S6). The root node of angiosperms was calibrated to 138.5 Mya (million years ago) based on estimates by Magallón et al. (2015). To assess the phylogenetic uncertainty in our dating estimates, we generated 1,000 bootstrap pseudo‐replicates using the best‐supported ML topology as constraint. Each pseudo‐replicate was individually dated using TreePL version 1.0, which is specifically designed to date large phylogenies (Smith & O’Meara, 2012). A small smoothing parameter of 0.003 was applied to minimize the influence of the large heterogeneity of substitution rates across the broad angiosperm phylogeny, while the default setting was kept for all other parameters (for more details to justify the smoothing parameter, see Janssens et al. (2020)). Changing the smoothing parameter to 1 or even 10 did not change the dating estimates for most of the clades studied (results not shown). The 1,000 dated trees were summarized into a single consensus tree to calculate the 95% range for the dating estimates of each node using TreeAnnotator version 1.8.4 with default settings (Drummond et al., 2012). The consensus tree is given in annotated nexus format and available on Dryad digital repository (https://doi.org/10.5061/dryad.kh189322s).

We present means and 95% ranges of stem and crown node estimates for colonization events of 24 lineages where transitions to insular woodiness occurred, and dating estimates for insular woody shifts of 23 lineages (*Aeonium* was excluded due to phylogenetic inconsistencies in the *Aeonium* alliance); 19 of these are considered equal since all island species are insular woody (Table S4). In addition, we added similar information for nine herbaceous, two ancestrally woody and four derived woody Canary lineages that acquired their (derived) woodiness before colonization on the archipelago (Table S4) and visualized all mean stem age estimates and their confidence ranges with respect to the palaeoclimatic timeline (Figure 2). All dating estimates are based on nodes identified in previous phylogenies, and the dates obtained for crown and stem node lineages were compared to published dates when available (Table S4).

## 3 RESULTS

### 3.1 Insular woodiness database

Our revised IW database based on the most recent phylogenies includes 196 insular woody species resulting from at least 35 independent shifts toward IW on the Canaries (compared to 220 insular woody species resulting from 38 shifts mentioned in Lens, Davin et al. (2013)). The updated phylogenetic insights suggest that an additional eight insular woody species could have evolved IW either on the Canaries or on nearby Macaronesian islands (species names highlighted in red in Table S1), which could further increase the number of insular woody shifts up to a maximum of 43 (Table S2). Out of the 196 species, 46% are native to the arid coastal areas and another 5% inhabit high altitude deserts above 2,000 m asl. Thirty‐nine percent occur in forests, with mesic laurel forests accounting for only 13% of the total number of insular woody species. Furthermore, rocks or cliffs are typical elements that occur in habitats of 43% of the species identified.

### 3.2 Palaeoclimate review for the Canary Islands

A detailed summary of the literature overview of the major palaeoclimatic events during the last 20 Myr for the Canary Islands, North Africa and the Mediterranean region, along with a few global trends, are presented in Table S3. The most important events that have impacted the palaeoclimate on the Canary Islands are visually summarized in Figure 2 and Figure S13. The two major palaeoclimatic events which have caused severe aridification episodes on the Canaries include (i) the Pleistocene fluctuations with drier glacial cycles of which the two main events date back 0.38–0.34 million years ago (Mya) and 0.48–0.42 Mya (Lisiecki & Raymo, 2005; Sánchez Goñi et al., 2016; Shackleton, 1987) and (ii) the onset of a Mediterranean seasonality on the Canaries with annual warm dry summers and mild wet winters (3.2 Mya) (Meco et al., 2011, 2015; Suc, 1984). Furthermore, the Messinian salinity crisis leading to a massive drying episode of the Mediterranean Sea (5.96–5.33 Mya) (Hsü et al., 1977; Jolivet et al., 2006), and the onset of desertification of Northern Africa (~7 Mya) (Schuster et al., 2006) have likely impacted the Canary Island vegetation, since most of the native Canary Island flora has its origin in the Mediterranean.

### 3.3 Molecular dating estimates

The colonization of the Canary Islands is inferred to be relatively recent for the clades studied, even with the most conservative dating estimates based on stem ages, with most events occurring in the last 3.2 million years (Myr) for both herbaceous and ancestrally or derived woody lineages (7/9 and 4/6 lineages for stem age estimates, respectively; Figure 2). With respect to dating events of the shifts toward IW, 14 of the 23 transitions studied (61%) have likely originated during the last 3.2 Myr, and 20 transitions (87%) are estimated to have occurred within the last 7 Myr, considering the large confidence intervals of the mean stem ages (Figure 2). The cumulative increase of insular woody shifts on the Canary Islands over time shows a peak from 4.2 Myr onwards (Figure 2). When crown node estimates are applied (Table S4 and Figure S13), the number of herbaceous and ancestrally/derived woody lineages originating during the last 3.2 Myr remain the same compared to stem node estimates, while the number of insular woody transitions increased to 65% and 96% during the last 3.2 and 7 Myr, respectively. The cumulative increase of insular woody shifts based on crown node estimates shows a peak from 4 Myr onwards (Figure S13).

## 4 DISCUSSION

To our knowledge, the presented updated insular woodiness (IW) review is the largest dating study of an insular flora, representing 94% of the insular woody species diversity on the Canary Islands. Using a critical re‐evaluation on the occurrence of species that have evolved their woodiness on the Canary Islands, we have identified 196 insular woody species that are the result of at least 35 independent shifts, many of which have originated in the last 3.2 Myr (Figure 2). This emphasizes again that the Canary Islands are a hotspot of IW (Lens, Davin et al., 2013).

The reason why a considerable number of species evolved into insular woody shrubs or trees following colonization of the Canaries remains incompletely understood. Based on their current distribution—50% of the extant insular woody Canary Island species are native to markedly dry (often coastal) regions (Table S1)—it is plausible that drought could have been a potential driver of wood formation in many insular woody lineages. To explore this drought hypothesis in more detail, we provided dating estimates of most insular woody lineages, embedded these estimates into a palaeoclimatic context (Figure 2 and Figure S13; for more details see Table S3), and interpreted the potential impact of palaeodrought on IW in a critical way.

Notably, our large‐scale two‐gene dating analysis shows that 70% of the dating estimates match previous studies, despite the use of alternative calibration points (e.g., different fossils, age of islands, or different secondary calibration points) and different DNA markers (García‐Verdugo et al., 2019; Kim et al., 2008; Kondraskov et al., 2015) (Table S4). Furthermore, we find that most Canary Island lineages studied are recent and date back to the late Pliocene and Pleistocene (Figure 2), in agreement with the review by García‐Verdugo et al. (2019), and thus further highlighting the frequent young age of many native lineages. This means that our dating approach represents a useful tool to answer broad‐scale evolutionary questions, especially when there are no fossil calibration points that could be assigned to the lineages of interest (see also Dagallier et al., 2020).

### 4.1 Repeated evolutionary transitions toward derived woodiness are recent and consistent with palaeoclimatic aridification events on the Canary Islands

We show that 18 out of 23 (78%) of the insular woody lineages originated from the early Pliocene onwards (Figures 2 and S13). In this Pliocene‐Pleistocene epoch, directly following the Messinian salinity crisis (5.96–5.33 Mya) and coinciding with a strong cumulative increase of insular woody shifts on the Canary Islands over time (Figure 2), a number of important palaeoclimatic aridification events took place on the Canaries: (a) the onset of the Mediterranean climate with annual warm summer droughts starting 3.2 Mya, likely caused by the redirection of oceanic currents of the Atlantic Ocean after the closing of the Isthmus of Panama (Haywood et al., 2000; Jiménez‐Moreno et al., 2010; Meco et al., 2015), and (b) the drier glacial periods during the Pleistocene, in particular two glacial maxima during 0.48–0.42 Mya and 0.38–0.34 Mya (Figure 2). These two short glaciation events are considered the most severe glacial periods across the Northern Hemisphere over the last 2 million years and are defined by the marine isotope stages (MIS) 12 and 10, respectively (Lisiecki & Raymo, 2005; Sánchez Goñi et al., 2016; Shackleton, 1987).

The colonization estimate of the insular woody *Argyranthemum* based on mean stem age (0.2 Mya, 95% range: 0.5–0.03 Mya) is consistent with MIS 10, which may imply that this relatively short but intense aridification episode could have driven woodiness and subsequent fast radiation in this genus, originating 19 Canary Island species. Without implying a causal relationship, the experimental study by Dória et al. (2018) also showed that the stems of the woody *Argyranthemum* species are better able to avoid drought‐induced gas embolisms inside water‐conducting vessels compared to their herbaceous relatives. This allows woody plants to survive by keeping their water flow column intact, while herbaceous plants will suffer from lethal levels of hydraulic failure at the same level of drought stress. Even if *Argyranthemum* was older (1.5–3.0 Mya based on divergence times from isozymes and from chloroplast restriction site DNA and a different outgroup sampling (Francisco‐Ortega et al., 1995; Francisco‐Ortega et al., 1997; Francisco‐Ortega et al., 1996; Table S4)), the same link between IW and palaeodrought can be established.

When zooming out to older geological time frames, our dating results based on stem or crown age estimates indicate that the evolution toward woodiness in respectively 87% and 96% of the insular woody clades studied likely originated during the last 7 Myr (Figures 2 and S13). The late Miocene is characterized by two major aridification events that happened in close proximity to the Canaries: (a) the onset of desertification of Northern Africa (~7 Mya) and (b) the Messinian salinity crisis leading to a massive drying event of the Mediterranean Sea due to closure of the Strait of Gibraltar (5.96–5.33 Mya). These two aridification events outside the Canaries likely impacted the vegetation on the archipelago, both because of its proximity to the mainland, and of the prevalence of north‐eastern trade winds that largely explains the Mediterranean origin for most of the native Canary Island lineages.

### 4.2 The observed pattern between insular woodiness and palaeodrought: identifying pitfalls

When tracing woodiness shifts on the Canaries back to the palaeoclimate in which they evolved, several sources of uncertainty must be accounted for. First and foremost, we show that most native Canary Island lineages, including insular woody but also derived woody, ancestrally woody and herbaceous lineages, have originated during the last 3.2 Myr (Figure 2). The review study by García‐Verdugo et al. (2019) confirmed the young age of many native Canary Island lineages, with mean crown node ages dating back 2.1 ± 2.4 Mya. There are two main reasons associated with the predominantly young age of the Canary Island flora: (1) the proximity of the North African coast and the species‐rich Mediterranean region, enabling a large number of colonization events facilitated by the north‐eastern trade winds, and (2) the high extinction rates typical of islands, coupled with severe changes in the archipelago's geo‐ecological complexity throughout its evolutionary history (Caujapé‐Castells et al., 2017; García‐Verdugo et al., 2019). Bearing this in mind, the observed pattern between woodiness and palaeodrought in recent geological times could be simply the result of chance, as a consequence of the high taxon turnover for the Canary Island flora (Linder, 2008).

Secondly, the estimated ages obtained for the insular woody clades are accompanied by large confidence intervals, which could make the link to a specific palaeoclimatic episode blurry (Figure 2). The power of the present study, however, is that we provide age estimates for 39 independent lineages that are directly comparable, as these are based on a single analysis with identical settings.

A third point of concern reflects the uncertainty regarding the ancestral habitats of insular woody lineages on a given island. Especially for high elevation islands, there is a large altitudinal range in vegetation types ranging from dry coastal scrub vegetation to wet higher‐elevation laurel forests (del‐Arco et al., 2009). Furthermore, the archipelago's geo‐ecological complexity has changed dramatically through time, with great impacts on the distribution and genetic makeup of the flora (Caujapé‐Castells et al., 2017). In this context, inferring the specific palaeoclimatic conditions for any population that colonized the Canaries in the past is challenging. One way to estimate the ancestral habitat is to perform ancestral area reconstructions based on species dense phylogenies representing (nearly) all current‐day species. Interestingly, the dry lowland scrub has been estimated to be the ancestral Canary habitat for the insular woody lineages of *Crambe* (Francisco‐Ortega et al., 2002), *Descurainia* (Goodson et al., 2006), *Echium* (García‐Maroto et al., 2009), and *Lotus* section *Pedrosia* (Jaén‐Molina et al., 2021).

### 4.3 Extreme case of rapid evolution toward insular woodiness: walking stick cabbage

It is hard to estimate how much time an herbaceous colonizer requires to evolve into an insular woody species. The very young age of some of the insular woody lineages studied here suggests that the transition from the herbaceous toward the woody growth form can evolve very rapidly (e.g., *Lotus*: 0.31–0 Mya; see also Jaén‐Molina et al. (2021)). Indeed, one of the most intriguing examples of a rapid IW evolution can be found on the Canary Islands, where humans introduced *Brassica oleracea* (cabbage), leading to the spectacular woody, ‘walking stick’ accession (Lens, Davin et al., 2013). The exact timing of this human introduction remains unclear: There is no archeological evidence of the presence of cabbage in the precolonial period from 3rd century BC to 15th century AC (Morales et al., 2009; Morales Mateos et al., 2017), although there is one reference quoting orchards of cabbages cultivated on the islands in 1,341 AC (Berthelot & Barker‐Webb, 1842). This means that cabbages could have been introduced roughly 700 years ago at the end of the precolonial period via trade with the Florentines, Genoese, Spaniards, and Portuguese. Therefore, it is plausible that a number of herbaceous Canary Island colonizers may have undergone major evolutionary changes toward the insular woody habit in a time window of only a few hundred or thousand years after their arrival.

### 4.4 Young but diverse: nearly all species‐rich genera on the Canaries include insular woody species

Insular woody genera are among the largest angiosperm genera native to the Canary Islands. Out of the top ten most species‐rich genera native to the Canary Islands, viz. *Aeonium* (28 sp.), *Sideritis* (25 sp.), *Limonium* (22 sp.), *Echium* (21 sp.), *Micromeria* (21 sp.), *Argyranthemum* (23 sp.), *Cheirolophus* (18 sp.), *Lotus* (19 sp.), *Sonchus* (18 sp.), and *Crambe* (12 sp.), eight include multiple insular woody species accounting for 143 out of 196 insular woody species on the Canary Islands (73%). In contrast, the herbaceous genera mostly do not harbor more than five species (*Silene* is a noteworthy exception; Arechavaleta et al., 2010). In other words, in situ wood development on the Canaries may have boosted species diversification.

Although the link between IW and diversification on the Canaries is obvious, identifying the traits that promote diversification is one of the unresolved key questions in plant evolutionary sciences (Patiño et al., 2017; Sauquet & Magallón, 2018) and goes beyond the scope of this study. The potential drivers behind species radiation are the result of complex ecological relationships and probably involve a range of abiotic (soils, climate, geology) and/or biotic traits (flower, seed, fruit, ploidy) that may or may not have co‐evolved with the insular woody life form and are perhaps more likely to cause geographic (and thus reproductive) isolation leading to speciation (Caujapé‐Castells, 2017; Givnish, 2010). The positive link between IW and diversification seems to contradict outcomes of large‐scale phylogenetic studies showing that herbaceous lineages have three to four times as many species as their (ancestrally) woody sister lineages, which likely reflects the shorter generation time of herbs often leading to significantly higher rates of molecular evolution compared to tall trees (Givnish, 2010; Smith & Donoghue, 2008). Since it is plausible that rates of molecular evolution between *insular* woody lineages (mostly comprised of small shrubs) and their herbaceous continental relatives (including many perennial herbs) resemble each other more, the increased diversification in many insular woody Canary Island lineages may not be in conflict with information in the literature.

In contradiction to several young, species‐rich insular woody genera, it is noteworthy that some of the older insular woody lineages have far fewer species (e.g., *Ixanthus*, 1 sp.; *Lavandula*, 5 sp). This is in conflict with the niche pre‐emption hypothesis stating that early colonizers have had more opportunity to occupy the available niches compared to later colonizers, and therefore, older lineages should have had more chance to diversify into larger clades (Silvertown, 2004; Silvertown et al., 2005). This paradox can be explained by the typical high taxon turnover of the native Canary Island flora (García‐Verdugo et al., 2019; Linder, 2008).

In conclusion, our results are consistent with palaeoclimatic aridification as a potential driver of woodiness that gave rise to 94% of the current diversity of insular woody Canary Island species as included in our study. However, our dataset does not allow us to test whether or not the observed predominant pattern of insular woody species having evolved in recent geological times characterized by dry palaeoconditions could simply be caused by a high taxon turnover, assisted by high extinction rates and frequent colonization from nearby continental sources. Likewise, uncertainties in dating estimates—common in any dating study—and marked habitat diversity in a geologically dynamic Canary Island setting further complicate the ability to assess whether palaeodrought was responsible for the repeated woodiness transitions that often gave rise to spectacular radiations. Bearing this in mind, it is unlikely that there is a single driver triggering wood formation across all the convergent evolutionary transitions. Although a majority of derived woody species across the world occur in regions with recurrent drought cycles, others are native to wet areas without drought (Frankiewicz et al., 2020; Kidner et al., 2016).

## CONFLICT OF INTEREST

The authors declare they have no conflict of interests.

## AUTHOR CONTRIBUTION

**Alexander Hooft van Huysduynen:** Data curation (equal); Formal analysis (lead); Investigation (equal); Methodology (equal); Visualization (lead); Writing‐original draft (lead); Writing‐review & editing (lead). **Steven Janssens:** Conceptualization (equal); Data curation (equal); Formal analysis (equal); Investigation (equal); Methodology (equal); Writing‐review & editing (supporting). **Vincent Merckx:** Formal analysis (equal); Methodology (equal); Supervision (supporting); Writing‐review & editing (supporting). **Rutger Vos:** Formal analysis (supporting); Methodology (supporting); Writing‐review & editing (supporting). **Luis Valente:** Formal analysis (supporting); Methodology (supporting); Writing‐review & editing (supporting). **Alexander Zizka:** Data curation (equal); Formal analysis (supporting); Methodology (supporting); Writing‐review & editing (supporting). **Maximilian Larter:** Formal analysis (supporting); Methodology (supporting); Writing‐review & editing (supporting). **Betül Karabayir:** Formal analysis (supporting); Investigation (equal); Methodology (supporting); Writing‐review & editing (supporting). **Daphne Maaskant:** Formal analysis (supporting); Investigation (equal); Methodology (supporting). **Youri Witmer:** Formal analysis (supporting); Investigation (equal); Methodology (supporting). **José María Fernández‐Palacios:** Formal analysis (supporting); Investigation (supporting); Methodology (supporting); Writing‐review & editing (supporting). **Lea de Nascimento:** Formal analysis (supporting); Investigation (supporting); Methodology (supporting); Writing‐review & editing (supporting). **Ruth Jaén‐Molina:** Investigation (equal); Writing‐review & editing (supporting). **Juli Caujapé Castells:** Investigation (equal); Writing‐review & editing (supporting). **Águedo Marrero‐Rodríguez:** Investigation (equal). **Marcelino del Arco:** Investigation (equal). **Frederic Lens:** Conceptualization (lead); Data curation (equal); Formal analysis (equal); Funding acquisition (lead); Investigation (equal); Methodology (lead); Project administration (lead); Supervision (lead); Writing‐original draft (lead); Writing‐review & editing (lead).

## Supporting information

Fig S1‐S12

Fig S13

Table S1‐S6

Supplementary Material

## Data Availability

DNA sequences available in GenBank under accession numbers: MN783748–MN783978. Mega phylogeny with dating estimation annotations available in the Dryad digital repository at https://doi.org/10.5061/dryad.kh189322s. Supplementary material (Tables S1–S6, Fig S1‐S13.) supporting this article have been uploaded in the Dryad digital repository at https://doi.org/10.5061/dryad.p5hqbzkq2.

## References

[ece37986-bib-0001] Arechavaleta, M., Rodriguez, S., Zurita, N., & Garcia, A. (2010). Lista de especies silvestres de Canarias. Hongos, plantas y animales terrestres. 2009 (pp. 1–579). Gobierno de Canarias.

[ece37986-bib-0002] Berthelot, S., & Barker‐Webb, P. (1842). Histoire Naturelle des Iles Canaries. Tome premier. Première partie. Contenant l’Ethnographie et les Annales de la Conquête. Bèthune [3 tomos, 8 vols.].

[ece37986-bib-0003] Böhle, U. R., Hilger, H. H., & Martin, W. F. (1996). Island colonization and evolution of the insular woody habit in *Echium* L. (Boraginaceae). Proceedings of the National Academy of Sciences, 93(21), 11740–11745. 10.1073/pnas.93.21.11740 PMC38128 8876207

[ece37986-bib-0004] Brodribb, T. (2019). The changing world of drought resistance. A commentary on: “Embolism resistance in stems of herbaceous *Brassicaceae* and *Asteraceae* is linked to differences in woodiness and precipitation”. Annals of Botany, 124(1), iv–v. 10.1093/aob/mcz110 PMC6676379 31373617

[ece37986-bib-0005] Brodribb, T. J., Powers, J., Cochard, H., & Choat, B. (2020). Hanging by a thread? Forests and drought. Science, 368(6488), 261–266. 10.1126/science.aat7631 32299945

[ece37986-bib-0006] Burns, K. C. (2019). Evolution in isolation: The search for an island syndrome in plants. Cambridge University Press.

[ece37986-bib-0007] Carlquist, S. J. (1974). Island biology. Columbia University Press. 10.5962/bhl.title.63768

[ece37986-bib-0008] Caujapé‐Castells, J., García‐Verdugo, C., Marrero‐Rodríguez, Á., Fernández‐Palacios, J. M., Crawford, D. J., & Mort, M. E. (2017). Island ontogenies, syngameons, and the origins and evolution of genetic diversity in the Canarian endemic flora. Perspectives in Plant Ecology, Evolution and Systematics, 27, 9–22. 10.1016/j.ppees.2017.03.003

[ece37986-bib-0009] Choat, B., Brodribb, T. J., Brodersen, C. R., Duursma, R. A., López, R., & Medlyn, B. E. (2018). Triggers of tree mortality under drought. Nature, 558(7711), 531–539. 10.1038/s41586-018-0240-x 29950621

[ece37986-bib-0010] Dagallier, L.‐P., Janssens, S. B., Dauby, G., Blach‐Overgaard, A., Mackinder, B. A., Droissart, V., Svenning, J.‐C., Sosef, M. S. M., Stévart, T., Harris, D. J., Sonké, B., Wieringa, J. J., Hardy, O. J., & Couvreur, T. L. P. (2020). Cradles and museums of generic plant diversity across tropical Africa. New Phytologist, 225(5), 2196–2213. 10.1111/nph.16293 PMC7027791 31665816

[ece37986-bib-0011] Darwin, C. (1859). On the origin of species by means of natural selection. J Murray.

[ece37986-bib-0012] Davidson, R., Vachaspati, P., Mirarab, S., & Warnow, T. (2015). Phylogenomic species tree estimation in the presence of incomplete lineage sorting and horizontal gene transfer. BMC Genomics, 16(Suppl 10), S1. 10.1186/1471-2164-16-S10 PMC4603753 26450506

[ece37986-bib-0013] del‐Arco, M. J., Rodríguez‐Delgado, O., Acebes, J. R., García‐Gallo, A., Pérez‐de‐Paz, P. L., González‐Mancebo, J. M., González‐González, R., & Garzón‐Machado, V. (2009). Bioclimatology and Climatophilous Vegetation of Gomera (Canary Islands). Annales Botanici Fennici, 46(3), 161–191. 10.5735/085.046.0301

[ece37986-bib-0014] Dória, L. C., Podadera, D. S., Arco, M., Chauvin, T., Smets, E., Delzon, S., & Lens, F. (2018). Insular woody daisies (Argyranthemum, Asteraceae) are more resistant to drought‐induced hydraulic failure than their herbaceous relatives. Functional Ecology, 32(6), 1467–1478. 10.1111/1365-2435.13085

[ece37986-bib-0078] Drummond, A. J., Suchard, M. A., Xie, D., & Rambaut, A. (2012). Bayesian phylogenetics with BEAUti and the BEAST 1.7. Molecular Biology and Evolution, 29(8), 1969–1973. 10.1093/molbev/mss075 22367748 PMC3408070

[ece37986-bib-0015] Doyle, J. A. (2012). Molecular and fossil evidence on the origin of angiosperms. Annual Review of Earth and Planetary Sciences, 40(1), 301–326. 10.1146/annurev-earth-042711-105313

[ece37986-bib-0016] Feild, T. S., Arens, N. C., Doyle, J. A., Dawson, T. E., & Donoghue, M. J. (2004). Dark and disturbed: A new image of early angiosperm ecology. Paleobiology, 30(1), 82–107. 10.1666/0094-8373(2004)030<0082:DADANI>2.0.CO;2

[ece37986-bib-0019] Francisco‐Ortega, J., Crawford, D. J., Santos‐Guerra, A., & Carvalho, J. A. (1996). Isozyme differentiation in the endemic genus *Argyranthemum* (Asteraceae: Anthemideae) in the Macaronesian Islands. Plant Systematics and Evolution, 202(3–4), 137–152. 10.1007/BF00983379

[ece37986-bib-0020] Francisco‐Ortega, J., Crawford, D. J., Santos‐Guerra, A., & Sa‐Fontinha, S. (1995). Genetic divergence among Mediterranean and Macaronesian genera of the subtribe Chrysantheminae (Asteraceae). American Journal of Botany, 82(10), 1321–1328. 10.1002/j.1537-2197.1995.tb12665.x

[ece37986-bib-0018] Francisco‐Ortega, J., Fuertes‐Aguilar, J., Kim, S.‐C., Santos‐Guerra, A., & Jansen, R. K. (2002). Phylogeny of the Macaronesian endemic Crambe section Dendrocrambe (Brassicaceae) based on internal transcribed spacer sequences of nuclear ribosomal DNA. American Journal of Botany, 89(12), 1984–1990. 10.3732/ajb.89.12.1984 21665627

[ece37986-bib-0017] Francisco‐Ortega, J., Santos‐Guerra, A., Hines, A., & Jansen, R. K. (1997). Molecular evidence for a Mediterranean origin of the Macaronesian endemic genus Argyranthemum (Asteraceae). American Journal of Botany, 84(11), 1595–1613. 10.2307/2446622 21708563

[ece37986-bib-0021] Frankiewicz, K. E., Oskolski, A., Banasiak, Ł., Fernandez, F., Reduron, J.‐P., Reyes‐Betancort, J.‐A., Szczeparska, L., Alsarraf, M., Baczyński, J., & Spalik, K. (2020). Parallel evolution of arborescent carrots *(Daucus)* in Macaronesia. American Journal of Botany, 107(3), 394–412. 10.1002/ajb2.1444 32147817 PMC7155066

[ece37986-bib-0022] Freiberg, M., Winter, M., Gentile, A., Zizka, A., Muellner‐Riehl, A. N., Weigelt, A., & Wirth, C. (2020). LCVP, The Leipzig catalogue of vascular plants, a new taxonomic reference list for all known vascular plants. Scientific Data, 7(1), 416. 10.1038/s41597-020-00702-z 33243996 PMC7693275

[ece37986-bib-0023] García‐Maroto, F., Mañas‐Fernández, A., Garrido‐Cárdenas, J. A., Alonso, D. L., Guil‐Guerrero, J. L., Guzmán, B., & Vargas, P. (2009). Δ6‐Desaturase sequence evidence for explosive Pliocene radiations within the adaptive radiation of Macaronesian *Echium* (Boraginaceae). Molecular Phylogenetics and Evolution, 52(3), 563–574. 10.1016/j.ympev.2009.04.009 19398027

[ece37986-bib-0024] García‐Verdugo, C., Caujapé‐Castells, J., & Sanmartín, I. (2019). Colonization time on island settings: Lessons from the Hawaiian and Canary Island floras. Botanical Journal of the Linnean Society, 191(2), 155–163. 10.1093/botlinnean/boz044

[ece37986-bib-0025] Givnish, T. J. (1982). On the Adaptive Significance of Leaf Height in Forest Herbs. The American Naturalist, 120(3), 353–381. 10.1086/283995

[ece37986-bib-0026] Givnish, T. J. (1998). Adaptive plant evolution on islands: Classical patterns, molecular data, and new insights. In P. Grant (Ed.), Evolution on islands (pp. 281–304). Oxford University Press.

[ece37986-bib-0027] Givnish, T. J. (2010). Ecology of plant speciation. Taxon, 59(5), 1326–1366. 10.1002/tax.595003

[ece37986-bib-0028] Goodson, B. E., Santos‐Guerra, A., & Jansen, R. K. (2006). Molecular systematics of *Descurainia* (Brassicaceae) in the Canary Islands: Biogeographic and taxonomic implications. Taxon, 55(3), 671–682. 10.2307/25065643

[ece37986-bib-0029] Haywood, A. M., Sellwood, B. W., & Valdes, P. J. (2000). Regional warming: Pliocene (3 Ma) paleoclimate of Europe and the Mediterranean. Geology, 28(12), 1063–1066. 10.1130/0091-7613(2000)28<1063:RWPMPO>2.0.CO;2

[ece37986-bib-0030] Helmus, M. R., Mahler, D. L., & Losos, J. B. (2014). Island biogeography of the Anthropocene. Nature, 513(7519), 543–546. 10.1038/nature13739 25254475

[ece37986-bib-0031] Hollingsworth, P. M., Forrest, L. L., Spouge, J. L., Hajibabaei, M., Ratnasingham, S., van der Bank, M., Chase, M. W., Cowan, R. S., Erickson, D. L., Fazekas, A. J., Graham, S. W., James, K. E., Kim, K.‐J., Kress, W. J., Schneider, H., van AlphenStahl, J., Barrett, S. C., van den Berg, C., Bogarin, D., … Little, D. P. (2009). A DNA barcode for land plants. Proceedings of the National Academy of Sciences, 106(31), 12794–12797. 10.1073/pnas.0905845106 PMC2722355 19666622

[ece37986-bib-0032] Hollingsworth, P. M., Graham, S. W., & Little, D. P. (2011). Choosing and using a plant DNA barcode. PLoS One, 10.1371/journal.pone.0019254 PMC3102656 21637336

[ece37986-bib-0033] Hooker, J. D. (1867). On insular floras: A lecture. Journal of Botany, 5, 23–31.

[ece37986-bib-0034] Hsü, K. J., Montadert, L., Bernoulli, D., Cita, M. B., Erickson, A., Garrison, R. E., Kidd, R. B., Mèlierés, F., Müller, C., & Wright, R. (1977). History of the Mediterranean salinity crisis. Nature, 267(5610), 399–403. 10.1038/267399a0

[ece37986-bib-0035] Izquierdo‐Carrasco, F., Smith, S. A., & Stamatakis, A. (2011). Algorithms, data structures, and numerics for likelihood‐based phylogenetic inference of huge trees. BMC Bioinformatics, 12(1), 470. 10.1186/1471-2105-12-470 22165866 PMC3267785

[ece37986-bib-0036] Jaén‐Molina, R., Marrero‐Rodríguez, Á., Caujapé‐Castells, J., & Ojeda, D. I. (2021). Molecular phylogenetics of *Lotus* (Leguminosae) with emphasis in the tempo and patterns of colonization in the Macaronesian region. Molecular Phylogenetics and Evolution, 154, 106970. 10.1016/j.ympev.2020.106970 33031929

[ece37986-bib-0037] Janssens, S., Couvreur, T. L. P., Mertens, A., Dauby, G., Dagallier, L.‐P., Vanden Abeele, S., Vandelook, F., Mascarello, M., Beeckman, H., Sosef, M., Droissart, V., van der Bank, M., Maurin, O., Hawthorne, W., Marshall, C., Réjou‐Méchain, M., Beina, D., Baya, F., Merckx, V., … Hardy, O. (2020). A large‐scale species level dated angiosperm phylogeny for evolutionary and ecological analyses. Biodiversity Data Journal, 8, e39677. 10.3897/BDJ.8.e39677 32015666 PMC6987248

[ece37986-bib-0038] Jiménez‐Moreno, G., Fauquette, S., & Suc, J.‐P. (2010). Miocene to Pliocene vegetation reconstruction and climate estimates in the Iberian Peninsula from pollen data. Review of Palaeobotany and Palynology, 162(3), 403–415. 10.1016/j.revpalbo.2009.08.001

[ece37986-bib-0039] Jolivet, L., Augier, R., Robin, C., Suc, J.‐P., & Rouchy, J. M. (2006). Lithospheric‐scale geodynamic context of the Messinian salinity crisis. Sedimentary Geology, 9(33), 9–32. 10.1016/j.sedgeo.2006.02.004

[ece37986-bib-0040] Katoh, K., & Standley, D. M. (2013). MAFFT multiple sequence alignment software version 7: Improvements in performance and usability. Molecular Biology and Evolution, 30(4), 772–780. 10.1093/molbev/mst010 23329690 PMC3603318

[ece37986-bib-0041] Kearse, M., Moir, R., Wilson, A., Stones‐Havas, S., Cheung, M., Sturrock, S., Buxton, S., Cooper, A., Markowitz, S., Duran, C., Thierer, T., Ashton, B., Meintjes, P., & Drummond, A. (2012). Geneious Basic: An integrated and extendable desktop software platform for the organization and analysis of sequence data. Bioinformatics, 28(12), 1647–1649. 10.1093/bioinformatics/bts199 22543367 PMC3371832

[ece37986-bib-0042] Kidner, C., Groover, A., Thomas, D. C., Emelianova, E., Soliz‐Gamboa, C., & Lens, F. (2016). First steps in studying the origins of secondary woodiness in Begonia (Begoniaceae): Combining anatomy, phylogenetics, and stem transcriptomics. Biological Journal of the Linnean Society, 117(1), 121–138. 10.1111/bij.12492

[ece37986-bib-0043] Kim, S.‐C., McGowen, M. R., Lubinsky, P., Barber, J. C., Mort, M. E., & Santos‐Guerra, A. (2008). Timing and tempo of early and successive adaptive radiations in Macaronesia. PLoS One, 3(5), e2139. 10.1371/journal.pone.0002139 18478126 PMC2367450

[ece37986-bib-0044] Kondraskov, P., Schütz, N., Schüßler, C., de Sequeira, M. M., Guerra, A. S., Caujapé‐Castells, J., Jaén‐Molina, R., Marrero‐Rodríguez, Á., Koch, M. A., Linder, P., Kovar‐Eder, J., & Thiv, M. (2015). Biogeography of mediterranean hotspot biodiversity: re‐evaluating the 'tertiary relict' hypothesis of macaronesian laurel forests. PLoS One, 10(7), e0132091– 10.1371/journal.pone.0132091 26173113 PMC4501571

[ece37986-bib-0045] Kress, W. J., & Erickson, D. L. (2007). A Two‐Locus Global DNA Barcode for Land Plants: The Coding rbcL Gene Complements the Non‐Coding trnH‐psbA Spacer Region. PLoS One, 2(6), e508– 10.1371/journal.pone.0000508 17551588 PMC1876818

[ece37986-bib-0046] Lens, F., Davin, N., Smets, E., & del Arco, M. (2013). Insular woodiness on the canary islands: a remarkable case of convergent evolution. International Journal of Plant Sciences, 174(7), 992–1013. 10.1086/670259

[ece37986-bib-0047] Lens, F., Eeckhout, S., Zwartjes, R., Smets, E., & Janssens, S. B. (2012). The multiple fuzzy origins of woodiness within Balsaminaceae using an integrated approach. Where do we draw the line? Annals of Botany, 109(4), 783–799. 10.1093/aob/mcr310 22190560 PMC3286280

[ece37986-bib-0048] Lens, F., Picon‐Cochard, C., Delmas, C. E. L., Signarbieux, C., Buttler, A., Cochard, H., Jansen, S., Chauvin, T., Chacon Doria, L., del Arco, M., & Delzon, S. (2016). Herbaceous angiosperms are not more vulnerable to drought‐induced embolism than angiosperm trees. Plant Physiology, 172(2), 661–667. 10.1104/pp.16.00829 27268961 PMC5047094

[ece37986-bib-0049] Lens, F., Tixier, A., Cochard, H., Sperry, J. S., Jansen, S., & Herbette, S. (2013). Embolism resistance as a key mechanism to understand adaptive plant strategies. Current Opinion in Plant Biology, 16(3), 287–292. 10.1016/j.pbi.2013.02.005 23453076

[ece37986-bib-0050] Linder, H. P. (2008). Plant species radiations: Where, when, why? Philosophical Transactions of the Royal Society B: Biological Sciences, 363(1506), 3097–3105. 10.1098/rstb.2008.0075 PMC2607312 18579472

[ece37986-bib-0051] Lisiecki, L. E., & Raymo, M. E. (2005). A Pliocene‐Pleistocene stack of 57 globally distributed benthic δ 18 O records. Paleoceanography, 20(1), 1–17. 10.1029/2004PA001071

[ece37986-bib-0052] Losos, J. B., & Ricklefs, R. E. (2009). Adaptation and diversification on islands. Nature, 457(7231), 830–836. 10.1038/nature07893 19212401

[ece37986-bib-0053] Magallón, S., Gómez‐Acevedo, S., Sánchez‐Reyes, L. L., & Hernández‐Hernández, T. (2015). A metacalibrated time‐tree documents the early rise of flowering plant phylogenetic diversity. New Phytologist, 207(2), 437–453. 10.1111/nph.13264 25615647

[ece37986-bib-0054] Meade, A., & Pagel, M. (2017). BayesTraitsV3. Available at http://www.evolution.rdg.ac.uk/BayesTraits.html

[ece37986-bib-0055] Meco, J., Koppers, A. A. P., Miggins, D. P., Lomoschitz, A., & Betancort, J.‐F. (2015). The Canary record of the evolution of the North Atlantic Pliocene: New 40 Ar 39 Ar ages and some notable palaeontological evidence. Palaeogeography, Palaeoclimatology, Palaeoecology, 435, 53–69. 10.1016/j.palaeo.2015.05.027

[ece37986-bib-0056] Meco, J., Muhs, D. R., Fontugne, M., Ramos, A. J., Lomoschitz, A., & Patterson, D. A. N. N. A. (2011). Late Pliocene and Quaternary Eurasian locust infestations in the Canary Archipelago. Lethaia, 44(4), 440–454. 10.1111/j.1502-3931.2010.00255.x

[ece37986-bib-0057] Miller, M. A., Pfeiffer, W., & Schwartz, T. (2010). Creating the CIPRES Science Gateway for inference of large phylogenetic trees. In 2010 Gateway Computing Environments Workshop (GCE) (pp. 1–8). 10.1109/GCE.2010.5676129

[ece37986-bib-0058] Morales, J., Rodríguez, A., Alberto, V., Machado, C., & Criado, C. (2009). The impact of human activities on the natural environment of the Canary Islands (Spain) during the pre‐Hispanic stage (3rd–2nd Century BC to 15th Century AD): An overview. Environmental Archaeology, 14(1), 27–36. 10.1179/174963109X400655

[ece37986-bib-0059] Morales Mateos, J., Rodríguez Rodríguez, A., & Henriquez Valido, P. (2017). Agricultura y recolección vegetal en la arqueología prehispánica de las Islas Canarias (siglos 111‐xv d.C.). In J. Fernández Eraso, J. A. Mujika Alustiza, A. Arrizabalaga Valbuena, & M. García Díez. Miscelánea en homenaje a Lydia Zapata Peña (1965‐2015) (pp. 189–218). Universidad del País Vasco.

[ece37986-bib-0060] Patiño, J., Whittaker, R. J., Borges, P. A. V., Fernández‐Palacios, J. M., Ah‐Peng, C., Araújo, M. B., Ávila, M. B., Cardoso, P., Cornuault, J., de Boer, E. J., de Nascimento, L., Gil, A., González‐Castro, A., Gruner, D. S., Heleno, R., Hortal, J., Illera, J. C., Kaiser‐Bunbury, C. N., Matthews, T. J., … Emerson, B. C. (2017). A roadmap for island biology: 50 fundamental questions after 50 years of The Theory of Island Biogeography. Journal of Biogeography, 44(5), 963–983. 10.1111/jbi.12986

[ece37986-bib-0061] Percy, D. M., & Cronk, Q. C. B. (1997). Conservation in relation to mating system in *Nesohedyotis arborea* (Rubiaceae), a rare endemic tree from St Helena. Biological Conservation, 80(2), 135–145. 10.1016/S0006-3207(96)00130-9

[ece37986-bib-0063] Sánchez Goñi, M. F., Llave, E., Oliveira, D., Naughton, F., Desprat, S., Ducassou, E., Hodell, D. A., & Hernández‐Molina, F. J. (2016). Climate changes in south western Iberia and Mediterranean Outflow variations during two contrasting cycles of the last 1Myrs: MIS 31–MIS 30 and MIS 12–MIS 11. Global and Planetary Change, 136, 18–29. 10.1016/j.gloplacha.2015.11.006

[ece37986-bib-0064] Sauquet, H., & Magallón, S. (2018). Key questions and challenges in angiosperm macroevolution. New Phytologist, 219(4), 1170–1187. 10.1111/nph.15104 29577323

[ece37986-bib-0065] Schuster, M., Duringer, P., Ghienne, J. F., Vignaud, P., Mackaye, H. T., Likius, A., & Brunet, M. (2006). The age of the Sahara desert. Science, 311(5762), 821. 10.1126/science.1120161 16469920

[ece37986-bib-0066] Shackleton, N. J. (1987). Oxygen isotopes, ice volume and sea level. Quaternary Science Reviews, 6(3–4), 183–190. 10.1016/0277-3791(87)90003-5

[ece37986-bib-0067] Silvertown, J. (2004). The ghost of competition past in the phylogeny of island endemic plants. Journal of Ecology, 92(92), 168–173. 10.1111/j.1365-2745.2004.00853.x

[ece37986-bib-0068] Silvertown, J., Francisco‐Ortega, J., & Carine, M. (2005). The monophyly of island radiations: An evaluation of niche pre‐emption and some alternative explanations. Journal of Ecology, 93(4), 653–657. 10.1111/j.1365-2745.2005.01038.x

[ece37986-bib-0069] Smith, S. A., & Donoghue, M. J. (2008). Rates of molecular evolution are linked to life history in flowering plants. Science, 322(5898), 86–89. 10.1126/science.1163197 18832643

[ece37986-bib-0070] Smith, S. A., & O’Meara, B. C. (2012). treePL: Divergence time estimation using penalized likelihood for large phylogenies. Bioinformatics, 28(20), 2689–2690. 10.1093/bioinformatics/bts492 22908216

[ece37986-bib-0071] Stamatakis, A. (2014). RAxML version 8: A tool for phylogenetic analysis and post‐analysis of large phylogenies. Bioinformatics, 30(9), 1312–1313. 10.1093/bioinformatics/btu033 24451623 PMC3998144

[ece37986-bib-0072] Stamatakis, A. (2016). The RAxML v8. 2. X Manual. Heidelberg Institute for Theoretical Studies. Available from: http://sco.h‐its.org/exelixis/web/software/raxml/#documentation

[ece37986-bib-0073] Suc, J. P. (1984). Origin and evolution of the mediterranean vegetation and climate in Europe. Nature, 307(5950), 429–432. 10.1038/307429a0

[ece37986-bib-0074] Thonglim, A., Delzon, S., Larter, M., Karami, O., Rahimi, A., Offringa, R., Keurentjes, J. J. B., Balazadeh, S., Smets, E., & Lens, F. (2020). Intervessel pit membrane thickness best explains variation in embolism resistance amongst stems of *Arabidopsis thaliana* accessions. Annals of Botany. 10.1093/aob/mcaa196 PMC8324034 33216143

[ece37986-bib-0075] Wallace, A. R. (1878). Tropical nature and other essays. Macmillan.

[ece37986-bib-0076] Whittaker, R. J., & Fernández‐Palacios, J. M. (2007). Island biogeography: Ecology, evolution, and conservation. Oxford University Press.

[ece37986-bib-0077] Whittaker, R. J., Fernández‐Palacios, J. M., Matthews, T. J., Borregaard, M. K., & Triantis, K. A. (2017). Island biogeography: Taking the long view of nature’s laboratories. Science, 357(6354), eaam8326. 10.1126/science.aam8326 28860356

