# Supplementary material for: Temporal and palaeoclimatic context of the evolution of insular woodiness in the Canary Islands
Source: Ecol Evol. 2021 Aug 17;11(17):12220–31. doi: 10.1002/ece3.7986 (PMC8427628; doi:10.1002/ece3.7986)

S1 *Argyranthemum*

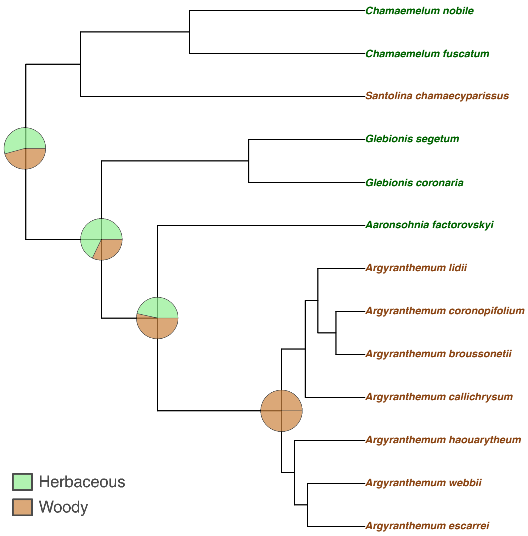

S2 *Carlina - Atractylis*

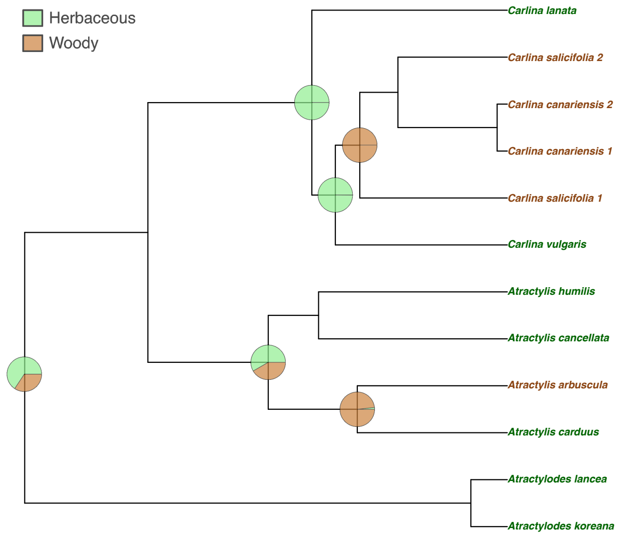

S3 *Convolvulus*

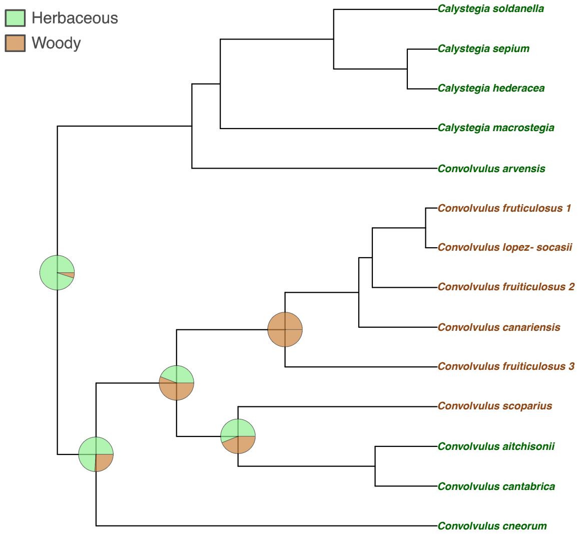

S4 *Crambe*

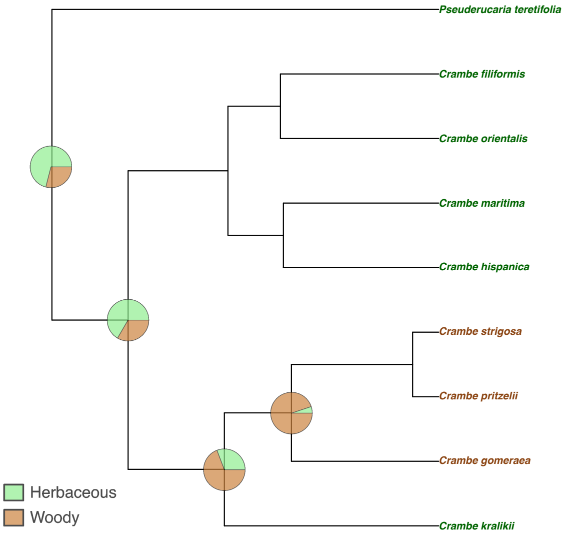

S5 *Descurania*

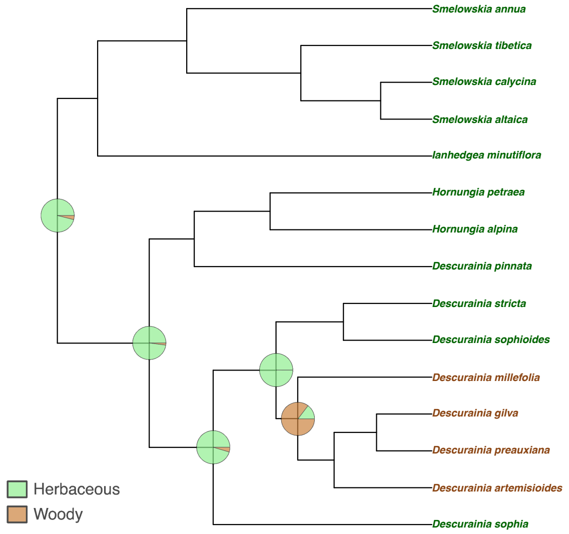

S6 *Digitalis - Plantago*

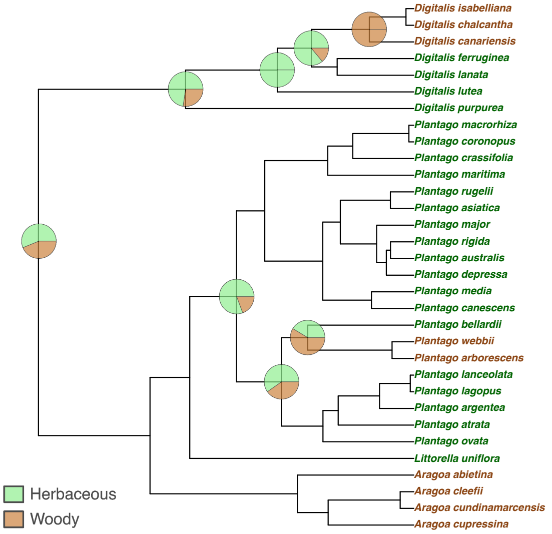

S7

Echium

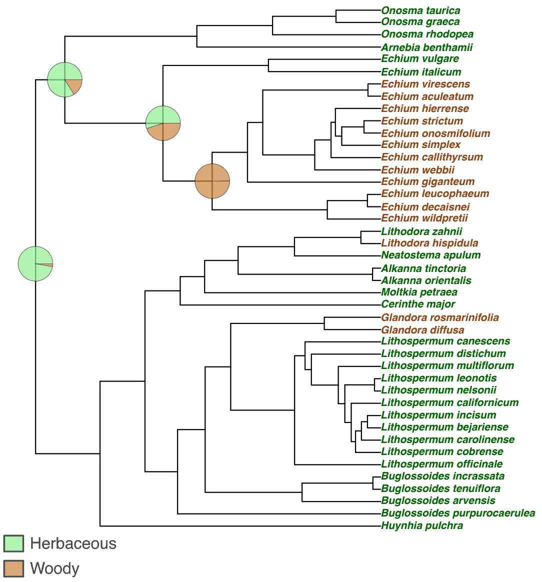

S8

Lobularia

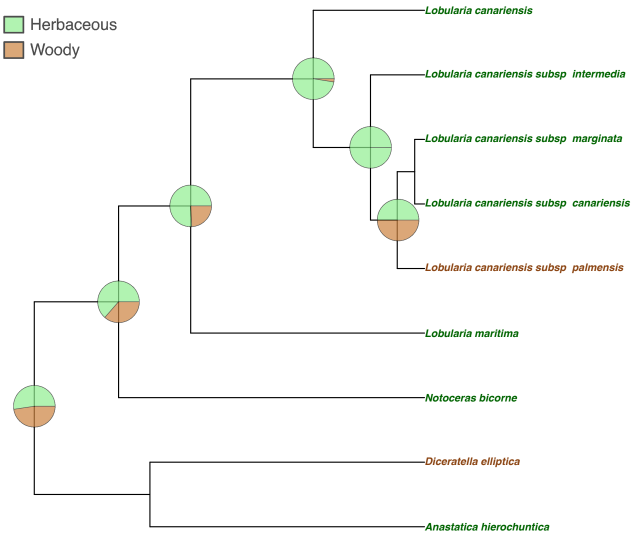

S9

Micromeria

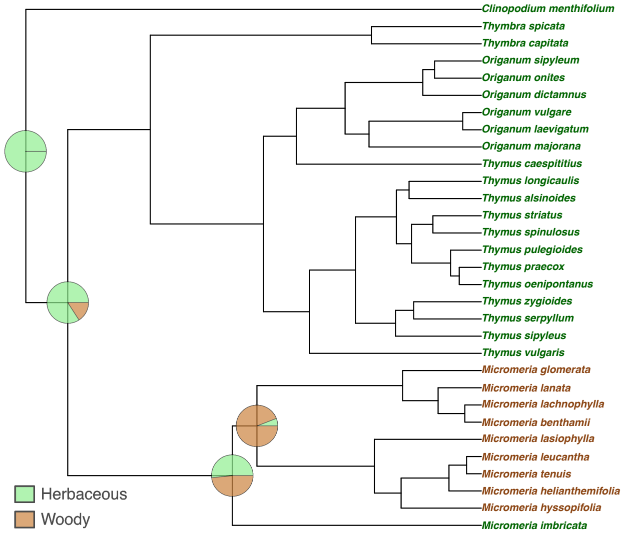

S10

Sideritis

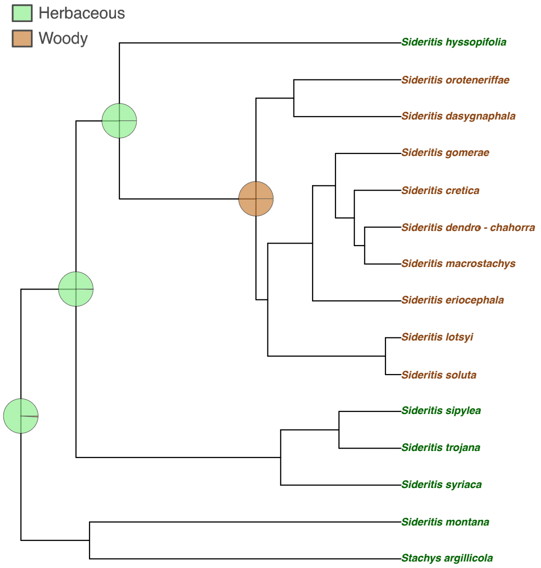

S11

Silene

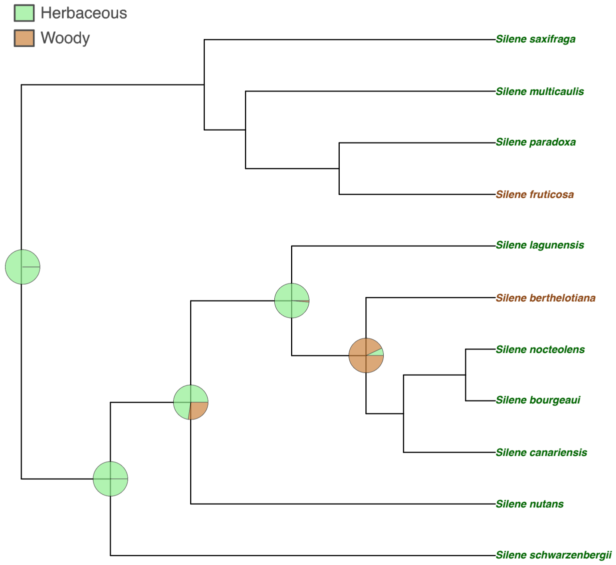

S12

Sonchus

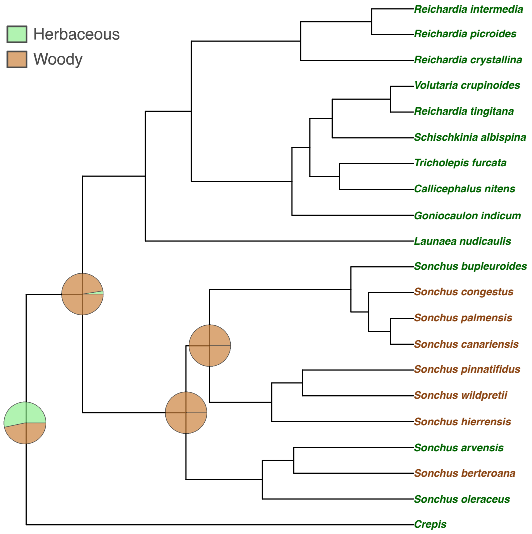

Supplement: Supplementary file 1 — Fig S1‐S12 [file ECE3-11-12220-s001.pdf]
