# Supplementary material for: Temporal and palaeoclimatic context of the evolution of insular woodiness in the Canary Islands
Source: Ecol Evol. 2021 Aug 17;11(17):12220–31. doi: 10.1002/ece3.7986 (PMC8427628; doi:10.1002/ece3.7986)
Supplement: Supplementary file 2 — Fig S13 [file ECE3-11-12220-s004.pdf]

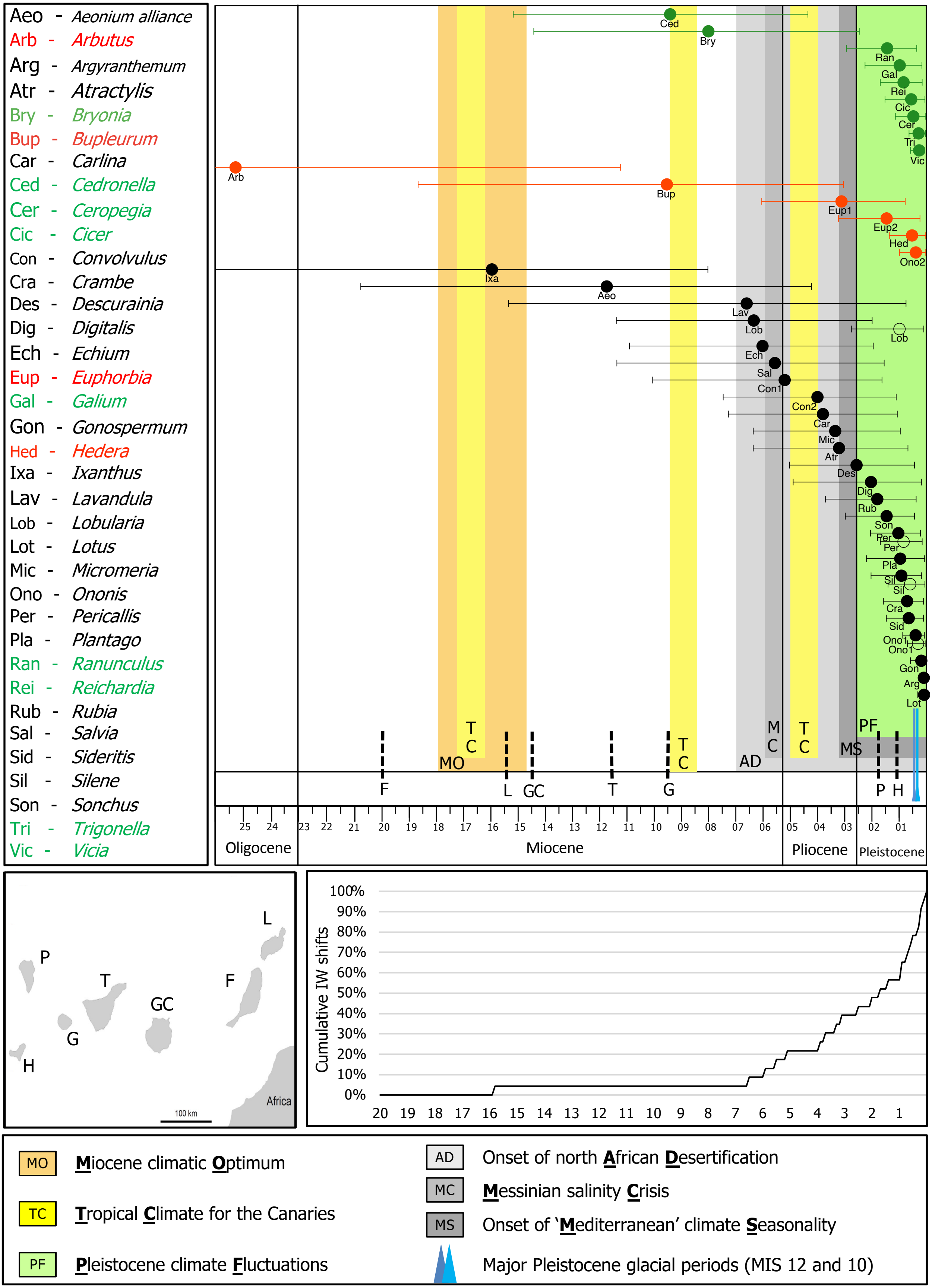

Aeo - Aeonium alliance
Arb - Arbutus
Arg - Argyranthemum
Atr - Atractylis
Bry - Bryonia
Bup - Bupleurum
Car - Carlina
Ced - Cedronella
Cer - Ceropegia
Cic - Cicer
Con - Convolvulus
Cra - Crambe
Des - Descurainia
Dig - Digitalis
Ech - Echium
Eup - Euphorbia
Gal - Galium
Gon - Gonospermum
Hed - Hedera
Ixa - Ixanthus
Lav - Lavandula
Lob - Lobularia
Lot - Lotus
Mic - Micromeria
Ono - Ononis
Per - Pericallis
Pla - Plantago
Ran - Ranunculus
Rei - Reichardia
Rub - Rubia
Sal - Salvia
Sid - Sideritis
Sil - Silene
Son - Sonchus
Tri - Trigonella
Vic - Vicia
Ced
Bry
Ran
Gal
Rei
Cic
Cer
Tri
Vic
Arb
Bup
Eup1
Eup2
Hed
Ono2
Ixa
Aeo
Lav
Lob
Lob
Ech
Sal
Con1
Con2
Car
Mic
Atr
Des
Dig
Rub
Son
Per
Per
Pla
Sil
Sil
Cra
Sid
Ono1
Ono1
Gon
Arg
Lot
TC
MO
TC
AD
MC
TC
MS
PF
F
L
GC
T
G
P
H
25 24 23 22 21 20 19 18 17 16 15 14 13 12 11 10 09 08 07 06 05 04 03 02 01
Oligocene
Miocene
Pliocene
Pleistocene
P
T
G
H
GC
F
L
Africa
100 km
Cumulative IW shifts
100%
90%
80%
70%
60%
50%
40%
30%
20%
10%
0%
20 19 18 17 16 15 14 13 12 11 10 9 8 7 6 5 4 3 2 1
MO Miocene climatic Optimum
TC Tropical Climate for the Canaries
PF Pleistocene climate Fluctuations
AD Onset of north African Desertification
MC Messinian salinity Crisis
MS Onset of 'Mediterranean' climate Seasonality
Major Pleistocene glacial periods (MIS 12 and 10)
