# Supplementary material for: Temporal and palaeoclimatic context of the evolution of insular woodiness in the Canary Islands
Source: Ecol Evol. 2021 Aug 17;11(17):12220–31. doi: 10.1002/ece3.7986 (PMC8427628; doi:10.1002/ece3.7986)
Supplement: Supplementary file 4 — Supplementary Material [file ECE3-11-12220-s003.pdf]

CAPTIONS FIGURES

Figure 1: *Explanatory graphic of ancestral woodiness (a), derived woodiness (b) and insular woodiness (c). The branches of the phylogenies are coloured according to growth form (red = woody; black = herbaceous). Star symbols highlight the estimated time of island colonisation and denote ancestral woody clades, derived woody clades , and insular woody clades in each panel, respectively.*

Figure 2: *Timeline of 39 Canary Island colonisation events and insular woody shifts based on conservative mean stem age estimates and their 95% range, along with the major palaeoclimatic events, ages of the individual Canary Islands over the last 26 Myr, and cumulative increase of insular woody shifts on the Canary Islands over the last 20 Myr. Black circles refer to the estimated mean ages for the timing of colonisation of the insular woody clades; for Lobularia, Ononis, Pericallis and Silene, which all include herbaceous as well as insular woody species on the Canary Islands, the insular woody shift occurred more recently in time (open circles). The time of colonisation of the herbaceous clades are illustrated in green and the time of colonisation of ancestrally woody clades and derived woody clades that have evolved their woodiness outside the archipelago are illustrated in red.*

SUPPORTING INFORMATION

Supporting information available for review at https://datadryad.org/stash/share/bhuTwk9YEZ5olESlF_JPtRctEUTplxWtgaTwe3cMV5U

Dated phylogeny available for review at https://datadryad.org/stash/share/S3zn4CYOuVZT7FJC6kDNDRn1zHPkmZiXKBsDS0BEzZU

Figure S1-S12: *Ancestral state reconstructions of Canary Island insular woody clades.*

Figure S13: *Timeline of 39 Canary Island colonisation events and insular woody shifts based on conservative mean crown age estimates and their 95% range, along with the major palaeoclimatic events, ages of the individual Canary Islands over the last 26 Myr, and cumulative increase of insular woody shifts on the Canary Islands over the last 20 Myr. Black circles refer to the estimated mean ages for the timing of colonisation of the insular woody clades; for Lobularia, Ononis, Pericallis and Silene, which all include herbaceous as well as insular woody species on the Canary Islands, the insular woody shift occurred more recently in time (open circles). The time of colonisation of the herbaceous clades are illustrated in green and the time of colonisation of ancestrally woody clades and derived woody clades that have evolved their woodiness outside the archipelago are illustrated in red.*

Table S1. Updated species database of 195 insular woody species native to the Canary Islands, along with information about phylogenetic position, maximum plant height, life form, geographic distribution, habitat, and elevation. The species highlighted in red represent eight additional insular woody species that developed their woodiness either on the Canary Islands or on nearby Macaronesian islands.

Table S2. Database of Canary Island genera where one or more insular woody shifts have been identified. Uncertainty in reported minimum number of IW transitions (trt_minshift_nr) is due to uncertainty with respect to uncertainties about phylogenetic resolution or geographical distrubition (IW transition on Canary Islands or other Macaronesian islands).

Table S3. Literature overview of palaeoclimatic events for the Canary Islands, northern Africa and the Mediterranean, and some global events during the archipelago formation.

Table S4. Summary of information for each Canary Island clade analysed. Mean molecular dating estimates and 95% confidence intervals of colonisation of the Canary clades and insular woody shifts are shown. Bootstrap support values for phylogenetic topology from 1000 pseudo-replicates are also given. Also, the number of Canary Island and insular woody species in each lineage is indicated, as well as dating estimates from previously published phylogenies and information regarding these estimates.

Table S5. List of species for which matK and rbcL sequences are generated, along with their growth form, voucher information, GenBank number and collection institute.

Table S6. List of calibration points and references used for dating the angiosperm wide phylogeny.
